# Internet Mindfulness Meditation Intervention for the General Public: Pilot Randomized Controlled Trial

**DOI:** 10.2196/mental.5900

**Published:** 2016-08-08

**Authors:** Helané Wahbeh, Barry S Oken

**Affiliations:** ^1^ Oregon Health & Science University Portland, OR United States

**Keywords:** Internet, mindfulness, meditation, behavior modification, controlled trial

## Abstract

**Background:**

Mindfulness meditation interventions improve a variety of health conditions and quality of life, are inexpensive, easy to implement, have minimal if any side effects, and engage patients to take an active role in their treatment. However, the group format can be an obstacle for many to take structured meditation programs. Internet Mindfulness Meditation Intervention (IMMI) is a program that could make mindfulness meditation accessible to all people who want and need to receive it. However, the feasibility, acceptability, and ability of IMMI to increase meditation practice have yet to be evaluated.

**Objectives:**

The primary objectives of this pilot randomized controlled study were to (1) evaluate the feasibility and acceptability of IMMIs in the general population and (2) to evaluate IMMI’s ability to change meditation practice behavior. The secondary objective was to collect preliminary data on health outcomes.

**Methods:**

Potential participants were recruited from online and offline sources. In a randomized controlled trial, participants were allocated to IMMI or Access to Guided Meditation arm. IMMI included a 1-hour Web-based training session weekly for 6 weeks along with daily home practice guided meditations between sessions. The Access to Guided Meditation arm included a handout on mindfulness meditation and access to the same guided meditation practices that the IMMI participants received, but not the 1-hour Web-based training sessions. The study activities occurred through the participants’ own computer and Internet connection and with research-assistant telephone and email contact. Feasibility and acceptability were measured with enrollment and completion rates and participant satisfaction. The ability of IMMI to modify behavior and increase meditation practice was measured by objective adherence of daily meditation practice via Web-based forms. Self-report questionnaires of quality of life, self-efficacy, depression symptoms, sleep disturbance, perceived stress, and mindfulness were completed before and after the intervention period via Web-based surveys.

**Results:**

We enrolled 44 adults were enrolled and 31 adults completed all study activities. There were no group differences on demographics or important variables at baseline. Participants rated the IMMI arm higher than the Access to Guided Meditation arm on Client Satisfaction Questionnaire. IMMI was able to increase home practice behavior significantly compared to the Access to Guided Meditation arm: days practiced (*P*=.05), total minutes (*P*=.01), and average minutes (*P*=.05). As expected, there were no significant differences on health outcomes.

**Conclusions:**

In conclusion, IMMI was found to be feasible and acceptable. The IMMI arm had increased daily meditation practice compared with the Access to Guided Meditation control group. More interaction through staff and/or through built-in email or text reminders may increase daily practice even more. Future studies will examine IMMI’s efficacy at improving health outcomes in the general population and also compare it directly to the well-studied mindfulness-based group interventions to evaluate relative efficacy.

**Trial Registration:**

Clinicaltrials.gov NCT02655835; http://clinicaltrials.gov/ct2/show/NCT02655835 (Archived by WebCite at http://www.webcitation/ 6jUDuQsG2)

## Introduction

Group mindfulness meditation interventions like Mindfulness-Based Stress Reduction (MBSR) and Mindfulness-Based Cognitive Therapy (MBCT) improve a variety of health conditions and quality of life, are inexpensive, easy to implement, have minimal if any side effects, and engage patients to take an active role in their treatment [1-4]. Despite the growing evidence for positive benefits from well-studied mindfulness-based programs like MBSR and MBCT, many people who could benefit from them face obstacles blocking their enrollment in and successful completion of the programs such as aversion to sharing, scheduling constraints, and travel and accessibility issues. First, the group classes require people to share in public (aversion to sharing). A certain percentage of people are fearful of social situations such as group or employee wellness programs. Statistics from the National Institute of Mental Health show that 18.1% US adults have an anxiety disorder annually (includes generalized, obsessive compulsive, panic, post-traumatic stress, social and agoraphobia) . Social phobias account for 6.8% of US adults over a lifetime [5]. These patients are likely averse to attending group sessions. The group classes also require attending at a specific time and day (scheduling constraints). Working adults may not have time to attend 2.5-hour sessions once a week for 8 weeks. In addition, the extensive home practice (45-60 minutes per day) was also a barrier to receiving group programs because many participants can not commit to home practice times or feel discouraged by their inability to maintain these practices times and thus, do not practice at all. Travel to a specific location requires time and transportation (travel and accessibility constraints). The travel time to get to the location where the class is being held is a burden, and it is not feasible for many, such as for those living in rural areas. This aversion to sharing, scheduling, and travel and accessibility factors are barriers for people who want and would benefit from the mindfulness interventions for reducing anxiety, depression, and pain.

An alternative delivery format may offer more options for people who want and need meditation therapy. In a cross-sectional Web-based survey, we asked 510 participants (mean age, 42 ± 15 years, 70% female) what format they would prefer to receive a mindfulness meditation intervention. The Internet received more positive responses than the group format, and 11% of participants said they would refuse a group format. The Internet was rated as the first choice format (Internet, 44%; individual, 37%; group, 19%), and group was the last choice for most participants (Internet, 29%; individual, 14%; group, 57%) [6]. The survey was administered on the Web, which could bias preference ratings toward the Internet version, but not explain the individual format being preferred to the group format. Regardless, these cross-sectional data lend support to the evaluation of different mindfulness meditation delivery formats.

Based on our laboratory experience with clinical mindfulness research studies and results from the cross-sectional survey, we transitioned from administering the standard group MBCT program to developing and then administering a one-on-one intervention. The intervention was adapted from MBCT and MBSR to include shorter weekly sessions (1 hour vs 2.5 hours), less weekly sessions (6 vs 8), shorter meditations (30 minutes vs 45 minutes) and still included all the core mindfulness concepts (see Wahbeh, 2014 for the full curriculum [4]). After using this program, we found that some participants, especially those with increased stress and life demands still had obstacles to receiving the one-on-one intervention. We then developed an Internet version of the one-on-one program that could be delivered on the Web at any time or place the participant could access the Internet [7]. Although the Internet version is not the full “dose” that a participant or patient might receive from MBSR or MBCT, it allows those who have obstacles to receiving the full programs to have some access to mindfulness meditation training that they would otherwise not have available to them or be willing to receive.

Internet formats of mindfulness meditation are promising because they address a number of barriers to care. They allow people to receive the therapy in the privacy of their homes, so there are no travel or accessibility constraints. Participants or patients complete the programs by themselves so there is no need to share sensitive or personal information in a group setting. Finally, people can take the program at any time, on any day so there are no scheduling constraints.

Internet meditation interventions have small but growing evidence for their use in a variety of settings. Studies have been conducted for a variety of populations: generally healthy adults [8], stressed older adults [7], smokers [9], and distressed cancer survivors [10]. They have also been examined for a variety of symptoms: anxiety [11], stress, anxiety and depression [12], stress [13], trauma [14], and residual depressive symptoms and relapse prophylaxis [15]. Most studies are small but show preliminary evidence for some benefit and no adverse events.

This study builds on this previous research of Web-based mindfulness meditation interventions and applies it to the general population, asking whether Web-based delivery programs can actually change behavior by increasing meditation practice compared with just having access to the guided meditations (GMs) themselves. The primary objectives of this pilot randomized controlled study were to (1) evaluate feasibility and acceptability of Internet Mindfulness Meditation Intervention or IMMI in the general population and (2) to evaluate IMMI’s ability to change meditation practice behavior. The secondary objective was to collect preliminary data on health outcomes.

## Methods

### Participants

Potential participants were screened by self-report to ensure appropriate enrollment according to the inclusion/exclusion criteria (Textbox 1). Broad inclusion criteria aided in recruitment and determine usage information from a wide variety of adults. To maximize the generalizability and public health relevance of the study, exclusion criteria were minimized and based primarily on screening out participants with an underlying illness that might limit the benefit of the intervention, confound outcomes, or increase the likelihood of dropout. Participants were recruited from the public through flyers, Web-based advertisements and listservs, the Oregon Health & Science University study board, and ResearchMatch [16]. The timeline for the project was as follows: funding – September 2014; recruitment – April 2015 to August 2015; closed to enrollment – September 2015; final data collection – December 2015.

**Inclusion and exclusion criteria.**

Inclusion criteria

- Age 18-80 years
- Access to computer and Internet
- Can hear and understand instructions
- Willing to accept randomization scheme and agrees to follow the study protocol

Exclusion criteria

- Significant acute medical illness that would decrease likelihood of study completion (self-report).
- Significant, untreated depression, as assessed by Center for Epidemiologic Studies Depression Scale-5 >20 during screening.
- Current daily meditation practice (≥5 min/day daily for at least 30 days in the last 6 months. Past practice not exclusionary but will be recorded)

### Study Procedures

This study was the first phase in a two-phase research program. Phase 1 is reported here. (For details of phase 2 see clinicaltrials.gov NCT02655835) The study was a pilot randomized controlled trial of English-speaking adults. The goal was to enroll at least 40 participants. All participants underwent a telephone screening, baseline measure collection, an intervention period, and end point measure collection. After the baseline measure collection, participants were randomized to 1 of 2 arms: IMMI or access to GM. The study was approved by the Oregon Health & Science University Institutional Review Board.

Following volunteer inquiries, the research assistant (RA) described the study, inclusion/exclusion criteria, risks and benefits of participation, and answered any questions by telephone. If the volunteer was still interested, the telephone screening was conducted by the RA, who was appropriately trained on study procedures, with an institutional review board–approved telephone screening script to confirm eligibility. The telephone screening script included the Center for Epidemiologic Studies Depression Scale-5 (CESD-5) [17] to rule out untreated depression (greater than 20). If the participant was not eligible based on these scores, the RA gave the volunteer resources for mental health care. If the participant was eligible, the RA continued with the assessment. Eligible and interested participants were sent a unique link to a Health Insurance Portability and Accountability Act–compliant SurveyMonkey website where they completed their baseline questionnaires. Due to the minimal risk of the study, a Waiver of Documentation of Consent was used. The first page of the SurveyMonkey [18] baseline questionnaire had language describing the nature of the study, risks and benefits of participation, voluntary participation, and the understanding that if the participant continues with the survey they are giving consent to have the information used for research purposes.

The baseline questionnaires were completed through the SurveyMonkey on the participants computer or an accessible computer to them before randomization. Self-report questions included demographics, quality of life (SF-36), self-efficacy (General Perceived Self-Efficacy Scale), depression symptoms (Center for Epidemiological Studies Depression Scale-20), sleep disturbance (Pittsburgh Sleep Quality Inventory), perceived stress (Perceived Stress Scale), and mindfulness (Five-Factor Mindfulness Questionnaire). All measures are widely used, validated, and sensitive to stress and/or mindfulness meditation and described in more detail in the Measures section.

Once participants completed their baseline surveys, they were randomized to the IMMI or the GM arm. Participants and study staff communicated via email and telephone. Participants received study staff emails and phone numbers in case they needed any assistance with technology or had questions about the content. The RA reminded all IMMI participants weekly to complete their sessions by email. Guided meditation participants were contacted weekly by email to report their adherence. Although in a group MBSR or MBCT program there would be intensive face-to-face support available for the participant in terms of problem-solving for their practice techniques and to answer questions about content, the goal for this study was to have very limited study staff interactions with the participants. This allowed us to evaluate IMMI independent from any teacher interaction or extensive study staff support.

IMMI is an interactive Web-based platform with one 60-minute session per week for 6 weeks with daily home practice between sessions. The IMMI program was accessed through the Internet. IMMI participants were emailed a link to the program and a unique username and password to enter the program and a digital workbook. IMMI is a standardized and structured program modeled after MBCT [19] and MBSR [20], 2 standardized, well-studied 8-week group programs that have strong evidence for their effectiveness [2]. The IMMI program was piloted in our laboratory with stressed older adults [7]. Content of the program was frozen for this trial. IMMI’s objectives are to (1) help participants understand their personal reactions to stress, (2) teach them skills to modify their stress reactions, and (3) promote their desire for self-care and feelings of competence and mastery. Each IMMI session included: (1) didactic instruction and discussion on stress, relaxation, meditation, and mind-body interaction; (2) instruction and practice in formal and informal mindfulness meditation; and (3) enquiry about problem-solving techniques regarding success and difficulty in practicing mindfulness (see Wahbeh, 2014 for full curriculum) [4]. The Mindful Body Scan and Sitting Meditation (awareness of breathing, body sensations, and cognitive and emotional processes) are some of the formal meditations. Observing and being mindful during daily activities like washing the dishes or making a cup of coffee are informal practices that are also taught to generalize mindfulness to daily life. Each week’s session had multiple lessons (6-10). Each session had multiple videos providing course instructions, content about a theme, and describing the home practice instructions. After each video, participants answered questions about the video content via the Web-based platform. The themes/lessons, in-session meditations, and home practice meditation recommendations are listed in Table 1. All meditations were guided and presented as an audio file accessed from within the IMMI program. Participants needed to complete each session and lesson to proceed to the next. Each week’s session began with a review of the previous week’s content and home-practice and ended with a summary of the current session and home practice recommendations for the following week.

**Table 1:** IMMI curriculum.

Week	Themes/Lessons	Meditations
1	Starting Where You are and Looking to the Future; What is Mindfulness?; Awareness Exercise; The Body Scan.	Awareness Exercise (I^a^ – 4 min); Body Scan (I – 30 min; H^b^ – daily)
2	The Body Scan; Dealing with Barriers; Responding versus Reacting; Staying Present: Different Objects of Focus; Breath is Life.	Body Scan (I – 30 min; H – daily); Sitting (I – 5 min; H – 10-15 min daily)
3	Sitting Meditation; Layers of Mindfulness and the Breathing Space; Caring for Ourselves.	Sitting (I – 30 min; H – daily); 3-step Breathing Space (I – 4 min; H – 3x/day)
4	Thoughts are Not Facts; Coping Space; Attitude of Acceptance; Mindfulness of Thoughts Meditation; Ways You Can See Your Thoughts Differently.	Sitting with Difficulty (I – 30 min; H – daily); Breathing Space (4 min; H – 3x/day); Mindfulness of Thoughts (I – 10 min)
5	Compassion Meditation; Practicing Compassion; Applying Compassion and 4-Step Breathing Space; 4-Step Coping Space; Giving Back; Taking Care of Ourselves.	Compassion (30 min; H – daily); 4-step Breathing Space; 4-step Coping Space (4 min; H – as needed)
6	Body Scan; Recap of Mindfulness Meditation; Life; The Future; Motivations; Everyday Usage of Mindfulness; Sitting Meditation.	Body Scan (I – 30 min); Sitting (I – 5 min); Home practice – Participant’s choice

^a^I: In-session meditations

^b^H: Home practice meditations

GM participants were emailed a link to access the same GMs used as home practice for IMMI (see Table 1). Participants accessed them as audio files on Dropbox.com. They were also emailed a brief handout about mindfulness meditation one time after learning about their randomization. The instructions for listening to the GMs were as follows, “Below you will find the links to the GMs. Feel free to listen to these directly from the link or download them to your device. You can listen to them as often as you would like.” Institutional affiliation was not displayed on either of the intervention platforms. Participants completed their end point questionnaires through the SurveyMonkey in the same manner as the baseline collection. In addition to the baseline measures, participants completed a Client Satisfaction Questionnaire (CSQ) to evaluate acceptability [21].

### Measures: Self-Rated Questionnaires Listed in Alphabetical Order

#### Center for Epidemiologic Studies Depression Scale

Depression was assessed during the screening procedure with a 5-item subset of the original 20-item scale (CESD-5). The CESD-5 raw score was multiplied by 4 for cutoff score criteria determination. The CESD-5 has demonstrated very good sensitivity (>0.84), specificity (≥0.80), and high validity (>0.90) for identifying people classified as depressed by the full 20-item scale [22]. The full version was used to evaluate depression symptoms at the baseline and end point visits. The CESD is a commonly used subjective measure of depressive symptoms. It asks participants about how they felt or behaved in the past week, yielding global scores ranging from 0-60, with higher scores indicating greater depression [23].

#### Client Satisfaction Questionnaire (CSQ-8)

The eight-item CSQ [21] was administered at the end point visit. It is an 8-item questionnaire used to assess satisfaction with the intervention. The questionnaire has demonstrated high internal consistency (α=.93) and strong construct validity, evidenced by correlation with service utilization and clinical outcomes [21]. The eight questions are presented in Table 2. Higher scores reflect greater satisfaction.

#### Credibility/Expectancy Questionnaire

The Credibility/Expectancy Questionnaire is a standardized instrument that assesses participant intervention expectancy and rationale credibility in clinical outcome studies [24]. The wording was minimally modified to assess attitudes toward the mindfulness interventions. The scale has a high internal consistency (α=.84) and good test-retest reliability (.75 credibility; .82 expectancy). Expectancy assessment is essential in controlled intervention studies [25].

#### Five-Factor Mindfulness Questionnaire (FFMQ)

Mindfulness was measured with the FFMQ, which assess 5 elements of a general tendency to be mindful in daily living: observing, describing, acting with awareness, nonjudging of inner experience, and nonreactivity to inner experience [26]. The questionnaire presents a series of 39 statements and asks participants to respond according to “what is generally true for you” using a Likert scale ranging from 1 (*never or very rarely true*) to 5 (*very often or always true*). The 5 facets can be combined to yield a composite score that reflects a global measure of mindfulness.

#### Pittsburgh Sleep Quality Index

Sleep quality and disturbances across a 1-month time span were measured with this 19-item instrument that yields 7 component scores: subjective sleep quality, sleep latency, sleep duration, habitual sleep efficiency, sleep disturbances, use of sleeping medication, and daytime dysfunction [27]. Only the sleep disturbance scores are reported in this paper missing data leading to the inability to calculate many of the subscales. Sleep quality suffers with chronic stress and is known to affect health and also be improved by mind-body interventions such as mindfulness meditation [28].

#### Perceived Stress Scale

Perceived stress was measured using the Perceived Stress Scale, a commonly used 10-item subjective instrument that measures respondents’ perceived stress in the past week [29]. It has good internal reliability (α=.76) and strong construct validity. The global score ranges from 0 to 36, with higher scores indicating greater perceived stress.

#### SF36v2

The SF36, version 2 is a 36-item self-report questionnaire of quality of life that measures eight health domains and results in a physical component score and mental component score. The SF Health Surveys are the most widely used tools in the world for measuring patient-reported outcomes, with more than 41,000,000 surveys taken and over 32,000 licenses issued to date [30].

Participant adherence for all participants was measured by subjective report of home practice. Participants were emailed a fillable form weekly to recall their daily practice for the previous week in minutes. Adherence was defined as the number of home-practice days, total number of minutes practiced over the six weeks, and average number of minutes practiced per practice day. For IMMI participants, the total number of sessions completed on the Web was also recorded.

### Statistical Analysis

Sample size was determined with power calculations using data from 40 participants who completed the same 6-week mindfulness meditation program as IMMI but in a one-on-one format. Daily meditation means and standard deviations during the 6-week program were used for the treatment values (mean, 28.3; standard deviation, 9.6) compared with the 6-week period after the intervention where they had access to the meditations but were not receiving any instruction (mean, 16.0; standard deviation, 13.1). Using a 2-tailed t-test model with an alpha of .05, 20 people in each group would result in power of 0.91. Dropouts were considered and with 15 completers in each group, power would be 0.81 [31].

Randomization was conducted by an unblinded RA with a covariate adaptive randomization approach [32] to help ensure arms are matched on age, gender, and depression score and to reduce selection bias after the baseline collection. The same RA enrolled and assigned participants to the interventions. Covariate adaptive randomization is recommended for small trials to balance important factors between groups [33]. Missing data were addressed at the participant level to minimize attrition and incomplete data. This was supported through mandatory fields in SurveyMonkey. Participants were considered “dropouts” if they completed fewer than half of the sessions (<3 of 6) and did not complete the end point collection [34]. Compliance enhancement measures included weekly check-ins by email. Statistical analysis was conducted in a blinded fashion with a blinded code for the intervention.

The primary and secondary aims were assessed as follows. The aim to evaluate feasibility and acceptability for IMMI was first analyzed in a descriptive fashion. Recruitment rates and drop-outs were described and noted for future studies and demographic data in relation to these numbers were qualitatively examined. The CSQ total and individual answers were then evaluated with 2-sample t-tests to evaluate differences between the arms. The aim to evaluate IMMI’s ability to change meditation practice behavior was analyzed as follows. Before inferential analysis, measure distribution was evaluated with Shapiro-Wilk test of normality. Expectancy for IMMI and GM, Physical Quality of Life scores, and Total Minutes practiced were not normally distributed. Nonparametric Kruskal-Wallis tests were conducted on these variables. Participant characteristics at baseline were evaluated for unbiased randomization with the *χ*^2^ test for discrete variables or the 2-sample Kruskal-Wallis test for non-normally distributed data. There were no unbalanced variables noted. A Wilcoxon paired test was conducted on expectancy for IMMI and GM for all participants. Differences between the IMMI and GM arms on all measures were evaluated with a simple 2-sample t-test (or nonparametric Kruskal-Wallis) for completers only.

The secondary aim to collect preliminary data on health outcome changes from IMMI was conducted in an exploratory fashion since the study was not powered to assess differences between the arms on these outcomes. Differences between the IMMI and GM arms on all measures were evaluated with a simple 2-sample t-test (or nonparametric Kruskal-Wallis) for completers only as described previously. Cohen’s d and 95% CIs on the change score from preintervention to postintervention was also calculated. Results from support power analyses and sample size estimation for future clinical trials examining these outcomes.

Statistical analyses were conducted in SPSS 20.0 (IBM, USA) and STATA 12.0 (Statacorp, LP, USA). This manuscript is reported according to the CONSORT statement for the reporting of randomized controlled trials [35] as well as the CONSORT-EHEALTH extension (see Multimedia Appendix 1). The study is registered at ClinicalTrials.gov (NCT02655835).

## Results

### Recruitment

**Figure 1:**
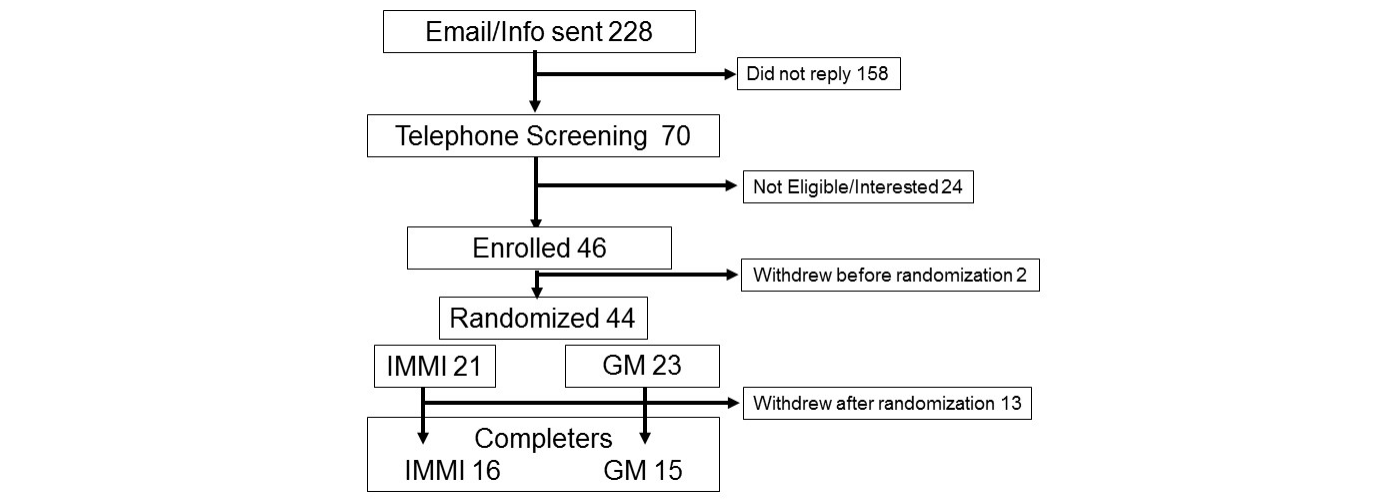
Recruitment.

An email invitation with information about the study was sent to 228 potential volunteers who responded to flyers or Web-based advertisements. Of those, 46 volunteers enrolled in the study. Most participants were from the Pacific Northwest (31, Oregon; 3, Washington). Other states represented were California (3), New York (3), Maryland (2), Massachusetts (1), Minnesota (1), and Virginia (1). Two withdrew before randomization. Thirteen withdrew or were lost-to-follow after randomization (5 were randomized to IMMI and 8 to GM). There were no demographic differences between participants who dropped out of the study and those who remained in the study (Figure 1). Participants reported no adverse events or side effects from either intervention.

Thirty-one participants were randomized and completed the study—16 in the IMMI arm and 15 in the GM arm. This reflects a 14% completion rate for those contacted and a 71% completion rate for those who were randomized. There was no difference in age, gender, education, annual household income, relationship status, health coverage, or experience with complementary and alternative medicine between the IMMI and GM arms (Table 2). Participants’ mean age was 42 ± 14 years, and they had an average of 18 ± 3 years of education. Participants were mostly Caucasian, employed, well-educated, females in a relationship, and with income level greater than $50,000. Of those unemployed, 17% from the GM arm were retired (0% from IMMI arm). Most people (except 9% in each arm) had some experience with complementary and alternative medicine.

At baseline, there were no differences between arms on physical or mental quality of life, depression symptoms, perceived stress, sleep disturbance, or mindfulness. Taking all participants together, there was a difference in expectancy (IMMI 7.24 ± 0.26; GM 6.78 ± 0.27; *Z*=2.06, *P*=.04) and credibility (IMMI 6.27 ± 0.30; GM 5.33 ± 0.35; *t(28)*=2.88, *P*=.008) (*Z*=2.86, *P*=.004 for the 2 arms with the IMMI intervention having higher perceived expectancy and credibility scores. These differences in perceived expectancy and credibility of the interventions were not evident between arms, namely all participants thought IMMI was a more credible intervention that would be more effective but these perceptions were the same across the 2 arms (Table 2).

**Table 2:** Demographics.

Demographics		GM^a^ (n=16)	IMMI^b^ (n=15)	Stats
Age (years)		45 (15)	38 (11)	*t*=91; *P*=.37
Gender				
	% Female	94	80	*X*^2c^=1.3; *P*=.25
Race				
	% Caucasian	88	86	*X*^2^=2.01; *P*=.57
	% Asian	0	7	
	% Hispanic	6	7	
	% Unknown	6	0	
Education				
	% Bachelor's or higher	81	87	*X*^2^=2.37; *P*=.67
Income				
	% 0-25K	19	13	*X*^2^=6.42; *P*=.49
	% 26-50K	25	34	
	% 50-100K	25	20	
	% >100K	31	33	
Employment status				
	% Employed	74	87	*X*^2^=3.40; *P*=.49
Relationship status				
	% In relationship	63	60	*X*^2^=5.79; *P*=.22
Health coverage				
	% With coverage	94	93	*X*^2^=.002; *P*=.96
Experience with CAM (%)				*X*^2^=7.03; *P*=.22
	None	6	7	
	Once in the past	0	7	
	Few times in the past	56	66	
	I go every few months	19	0	
	I go every month	6	20	
	I go every few weeks	13	0	
	I go more than once a week	0	0	
Expectancy^d^				
	IMMI	6.94 (.42)	7.62 (.26)	*X*^2^*=*.40, *P*=.52
	GM	6.61 (.37)	7.00 (.41)	*X*^2^=.66, *P*=.42
Credibility				
	IMMI	5.88 (.41)	6.76 (.43)	*t*=−1.48, *P*=.15
	GM	5.22 (.47)	5.47 (.53)	*t*=−.35, *P*=.73

^a^GM: access to guided meditation

^b^IMMI: Internet Mindfulness Meditation Intervention

^c^*X*^2^: Chi-square test for categorical variables

^d^Expectancy did not have normal distribution so a nonparametric Kruskal-Wallis chi-square test was used.

**Table 3:** Client Satisfaction Questionnaire.^a^

Items	GM^b^ (n=16)	IMMI^c^ (n=15)	Stats
1. How would you rate the quality of service you have received?	2.29 (0.91)	3.13 (0.83)	*t*= *−* 2.61, *P*=.02
2. Did you get the kind of service you wanted?	2.36 (0.63)	2.93 (0.88)	*t*= *−* 2.01, *P*=.05
3. To what extent has our program met your needs?	2.07 (0.83)	2.67 (0.90)	*t*=1.85, *P*=.08
4. If a friend were in need of similar help, would you recommend our program to him or her?	2.31 (0.95)	3.07 (0.88)	*t*= *−* 2.20, *P*=.04
5. How satisfied are you with the amount of help you have received?	2.00 (0.78)	2.87 (0.74)	*t*= *−* 3.06, *P*=.005
6. Have the services you received helped you to deal more effectively with your problems?	2.36 (0.50)	2.67 (0.62)	*t*=1.48, *P*=.15
7. In an overall, general sense, how satisfied are you with the service you have received?	2.29 (0.73)	3.00 (0.93)	*t*=2.30, *P*=.03
8. If you were to seek help again, would you come back to our program?	2.29 (0.73)	2.80 (0.77)	*t*= *−* 1.84, *P*=.08
Client satisfaction total	17.79 (1.31)	23.13 (1.25)	*t*= *−* 2.95, *P*=.007

^a^Values are means (standard deviation).

^b^GM: access to guided meditation

^c^IMMI: Internet Mindfulness Meditation Intervention

**Table 4:** Change in meditation practice behavior.

Item	GM^a^ (n=16)	IMMI^b^ (n=15)	Statistics
Days practiced over 6 weeks (total)	14.38 (2.67)	22.20 (2.92)	t^c^=−1.98, *P*=.05
Minutes practiced over 6 weeks (total)	176.25 (157.42)	507.00 (424.94)	*X*^2d^=6.4, *P*=.01
Average minutes per practice day	13.35 (1.45)	19.77 (2.97)	t=−1.98, *P*=.05
Weekly logs completed	3.44 (0.40)	4.30 (0.49)	t=−1.32, *P*=.20

^a^IMMI: Internet Mindfulness Meditation Intervention

^b^GM: Access to guided meditation

^c^*t*: Student’s t test

^d^*X*^2^: Kruskal-Wallis test

### Feasibility and Acceptability

On average and also by individual questions, participants in the IMMI arm rated the intervention higher than participants in the GM arm on the CSQ (Table 3).

### Change in Meditation Practice Behavior

The primary aim of the study was to evaluate IMMI’s ability to change meditation home practice behavior. The IMMI participants completed 4.27 ± 2.3 Web-based lessons (range 0-6). Eight participants completed all 6 Web-based lessons. IMMI had significantly more home practice days, total minutes practiced, and average minutes per day (Table 4). There was no difference between arms on weekly reporting of home practice.

**Table 5:** Preliminary data on health outcomes.

Variable	GM^a^ (n=16)			IMMI^b^ (n=15)			Statistic	Cohen’s D on Δ 95% CI
	Pre	Post	Δ	Pre	Post	Δ		
SF36-Physical	55.18 (7.67)	55.82 (9.78)	0.64 (3.53)	53.83 (6.57)	53.15 (7.06)	−0.68 (5.42)	*X*^2c^=0.83 *P*=.36	0.30 (−.42 to .99)
SF36-Mental	43.58 (11.18)	46.02 (10.92)	2.44 (8.95)	44.65 (9.17)	47.93 (9.44)	3.27 (5.43)	*t*^d^=−0.31 *P*=.76	−0.12 (−.82 to .59)
Depression (CESD^e^)	15.63 (7.29)	14.00 (6.76)	−1.36 (4.88)	15.73 (7.21)	14.27 (5.52)	−1.47 (6.39)	*t*=0.05 *P*=.96	0.02 (−.71 to .75)
Self-Efficacy (GPSE^f^)	31.00 (5.10)	32.86 (5.11)	1.29 (3.81)	32.07 (3.9)	32.93 (4.35)	0.87 (2.95)	*t*=0.33 *P*=.74	0.13 (−.61 to .85)
Perceived Stress (PSS^g^)	17.94 (7.58)	13.79 (6.61)	−3.36 (3.41)	16.53 (5.29)	14.67 (5.95)	−1.87 (5.19)	*t*=−.91 *P*=.37	0.22 (−1.1 to .40)
Sleep Disturbance (PSQI^h^)	1.06 (0.68)	0.88 (0.5)	−0.19 (0.75)	1.27 (0.59)	1.27 (0.46)	0 (0.53)	*t*=−.80 *p*=.43	−0.30 (−.99 to .42)
Mindfulness (FFMQ^i^)	124.13 (24.26)	132.5 (22.02)	7.21 (15.33)	128.47 (14.27)	131.8 (13.96)	3.33 (18.23)	*t*=0.62 *P*=.54	0.24 (−.50 to .96)

^a^GM: Access to guided meditation

^b^IMMI: Internet Mindfulness Meditation Intervention

^c^*X*^2^: Kruskal-Wallis test

^d^*t*: Student’s t test

^e^CESD: Center for Epidemiologic Studies Depression Scale

^f^GPSE: general perceived self-efficacy

^g^PSS: Perceived Stress Scale

^h^PSQI: Pittsburgh Sleep Quality Index

^i^FFMQ: Five-Factor Mindfulness Questionnaire

### Health Outcomes

Because this was a pilot study and not powered to evaluate superiority of the 2 arms, we did not expect to see significant findings in between-arm analysis. As expected, we did not see any differences (Table 5).

## Discussion

### Overview

This study was a pilot study examining the feasibility and acceptability of an Internet mindfulness meditation intervention for the general public, whether it could cause behavior change (ie, increase in daily meditation), and secondarily, collecting preliminary data on health outcomes. We found that an Internet mindfulness meditation intervention for the general public was feasible and acceptable and increased daily meditation compared with the control arm. Health outcome data were collected for future study preparation.

### Demographics

The covariate adaptive randomization was effective resulting in similar characteristics for both arms. The participants were mostly women, which is common for complementary and alternative medicine modalities [36]. Interestingly, most participants had only tried CAM a few times in the past. The racial distribution reflected the Portland, Oregon metropolitan area where most (74%) participants were from. Income distribution matched the United States where the mean household income, according to the US Census Bureau 2014 Annual Social and Economic Supplement, was $72,641 (median $51,939). Most participants were employed, and many of those not employed were retired. We had anticipated more unemployed participants since the intervention does require a time commitment, but this was not the case.

### Feasibility and Acceptability

Recruitment was feasible, with a 20% enrollment rate from those contacted and a 71% completion rate from enrollees. The participants found IMMI acceptable and more acceptable compared with the GM participants on the CSQ. Although the average scores for the total and individual questions were not excellent (ie, as high as the maximum value of 4), they were all above median of 2.5 reflecting average-to-good satisfaction. The interaction with study staff for this study was minimal. The RA contacted the participants by email once a week; to remind IMMI participants to complete sessions and collect adherence for the GM participants. The goal of this study was to have very limited interactions with the participants, and thus evaluate the program on its own without study staff support. Increased interaction with study staff may have improved the client satisfaction scores.

There was also a difference in the expectancy participants had toward the 2 interventions introducing an inherent bias into the study. Looking at expectancy and credibility scores of all participants before randomization, IMMI scored significantly higher than GM. The skewed bias toward IMMI was present in all participants before randomization and was not different between arms (ie, both arms felt IMMI was more credible and would be more effective). Recruitment materials attempted to be as unbiased as possible with language in regard to which arm would improve meditation practice. Regardless, the differences between the content of the arms were clear and reflected in the skewed expectancy and credibility.

### Change in Meditation Practice Behavior

Adherence was lower than we would have hoped. Participants completed about half of the weekly logs for home practice. Future studies will incorporate objective adherence built into the program so that we can track actual practice time more accurately as we have done in other studies [7,37,38]. The practice days were lower than we have seen in previous studies and only 8 IMMI participants completed all 6 Web-based sessions [7].

Regardless of this low compliance, IMMI did demonstrate the ability change behavior through increased meditation practice. IMMI participants had twice as many total meditation practice minutes over the 6 weeks. Most Americans have access to GMs through buying CDs, downloading from the Internet, or watching on YouTube. Considering the increasing positive evidence for meditation practice, encouraging daily meditation practice is a beneficial behavioral goal. Adding a Web-based meditation program can help encourage the daily meditation practice behavior. Although interactions with study staff and participants were purposefully kept to a minimum, programs that had more interaction and/or built in reminders through email or text to practice daily may result in an even greater level of daily practice.

### Health Outcomes

As expected, we found no differences between arms on health outcomes. The health outcome data will support planning for future clinical trials to evaluate the efficacy of IMMI in the general population.

### Limitations

This study addressed whether IMMI was feasible and acceptable to adults in the public, whether it increased meditation practice compared with access to GMs, and collected preliminary data on general health outcomes. The study was not powered to assess differences between the IMMI and the GM arm. From the effect sizes and large confidence intervals seen for the health outcomes, we are uncertain of any clinical effect. More research is needed to ascertain this. It may be that the intervention is more useful for participants with significant mental health symptoms rather than healthy adults in the public. Future studies would include or perhaps target those participants to evaluate IMMI with that population. In addition, this study found that IMMI was acceptable and feasible compared with a very low-dose mindfulness meditation. However, our results do not imply anything about how IMMI would compare with the well-studied group mindfulness-based meditation programs like MBSR and MBCT. One could consider IMMI an entry-level experience to mindfulness meditation, with a greater dose than simply having GMs but a lesser dose compared with MBSR and MBCT. Future studies could compare multiple delivery formats with different doses to each other to evaluate the effect of mindfulness dose on adherence and effects. In addition, the study was designed to assess shorter-term effects of mindfulness meditation (6 weeks). Ideally, a study would assess sustained effects over a longer period. The study used novel technology that may be difficult for some people to use and participants must have access to computers and Internet to be able to participate. The IMMI program is in an early pilot phase and has not been developed in languages for non–English-speaking individuals. Due to language and cultural differences, the tool cannot yet be developed for non–English-speaking individuals. Plans to develop such tools are contingent on showing efficacy in the general population.

### Conclusion

In conclusion, IMMI was found to be feasible and acceptable. IMMI was also able to change behavior and increase daily meditation practice compared with the GM arm control. More interaction through staff and/or through built-in email or text reminders may increase daily practice even more. Future studies will examine IMMI’s efficacy at improving health outcomes in the general population.

## Abbreviations

CESD: Center for Epidemiologic Studies Depression Scale
CSQ: Client Satisfaction Questionnaire
FFMQ: Five Factor Mindfulness Questionnaire
GM: access to guided meditations
HIPAA: Health Insurance Portability and Accountability Act
IMMI: Internet Mindfulness Meditation Intervention
IRB: institutional review board
MBCT: Mindfulness-Based Cognitive Therapy
MBSR: Mindfulness-Based Stress Reduction
RA: research assistant
SF-36: 36-Item Short Form Health Survey

## Appendix

Multimedia Appendix 1 CONSORT eHealth Checklist.
